# Differential ageing trajectories in motivation, inhibitory control and cognitive flexibility in Barbary macaques (*Macaca sylvanus*)

**DOI:** 10.1098/rstb.2019.0617

**Published:** 2020-09-21

**Authors:** Eva-Maria Rathke, Julia Fischer

**Affiliations:** 1 Cognitive Ethology Laboratory, German Primate Center, Leibniz Institute for Primate Research, Kellnerweg 4, 37077 Göttingen, Germany; 2 Department for Primate Cognition, Georg-August-University Göttingen, Göttingen, Germany; 3 Leibniz ScienceCampus Primate Cognition, Göttingen, Germany

**Keywords:** ageing, motivation, persistence, inhibitory control, cognitive flexibility

## Abstract

Across the lifespan, the performance in problem-solving tasks varies strongly, owing to age-related variation in cognitive abilities as well as the motivation to engage in a task. Non-human primates provide an evolutionary perspective on human cognitive and motivational ageing, as they lack an insight into their own limited lifetime, and ageing trajectories are not affected by customs and societal norms. To test age-related variation in inhibitory control, cognitive flexibility and persistence, we presented Barbary macaques (*Macaca sylvanus*), living at La Forêt des Singes in Rocamadour (France), with three problem-solving tasks. We conducted 297 trials with 143 subjects aged 2–30 years. We found no effect of age on success and latency to succeed in the inhibitory control task. In the cognitive flexibility task, 21 out of 99 monkeys were able to switch their strategy, but there was no evidence for an effect of age. Yet, the persistence in the motivation task as well as the overall likelihood to participate in any of the tasks declined with increasing age. These results suggest that motivation declines earlier than the cognitive abilities assessed in this study, corroborating the notion that non-human primates and humans show similar changes in motivation in old age.

This article is part of the theme issue ‘Evolution of the primate ageing process'.

## 1. Introduction

Owing to the growing proportion of older persons in human societies, research on the determinants of ageing and their consequences on social life, physical and mental health is gaining increasing attention [1]. One research focus is on the impact of ageing on cognitive processes that fall under the umbrella term ‘executive functions'. Such processes include inhibitory control and cognitive flexibility. Inhibitory control can be defined as overcoming an impulse or automatic response in order to change to a more appropriate and goal-oriented behaviour [2]. Cognitive flexibility is based on the ability to discard a non-functional strategy and shift to a novel adaptive one, and can be conceived as a prerequisite for flexible behaviour [3]. Inhibitory control and cognitive flexibility belong to a multidimensional complex of cognitive processes that regulate the behaviour of an individual in the environment [4]. Both inhibitory control and cognitive flexibility may themselves be supported by different cognitive processes [5].

According to the *inhibitory deficit hypothesis*, difficulties in the allocation of attention contribute to cognitive performance deficits with increasing age. Older persons have been found to exhibit more problems suppressing distracting as compared with relevant information [2]. There are, however, studies that find no such deficits with age. These studies challenge a general age-related decline in inhibitory control and rather suggest task-specific inhibitory impairment [6]. For instance, inhibitory control deficits appear more likely to be related to motor responses rather than attention span. Tasks that require inhibiting a behavioural response showed an age-related decrease [7]. Similarly, the capacity for flexible behaviour was reported to decrease with age. Cognitive flexibility can be measured in various ways, for instance assigning items to various categories (task switching). In study designs where participants had to switch between different categorization rules, older adults produced more errors and showed higher switching costs (i.e. reaction times) compared with younger adults [8].

For a full comprehension of the trajectory of cognitive processes, it is essential to consider the entire lifespan. For instance, inhibitory control appears to follow a U-shaped curve. Inhibitory capacities require time to develop as the involved brain regions, predominantly the prefrontal cortex, require time to mature up into adolescence and early adulthood. From adulthood on, the brain undergoes structural changes, with an increased propensity for atrophy of the prefrontal cortex [9]. These changes have been linked to an impaired cognitive performance such as a reduced ability to inhibit prepotent movements or decisions [10].

Cognitive performance depends not only on the ability to understand and solve a task, but also the motivation to engage in such a task, and to persevere when the solution does not immediately become clear. One of the core assumptions in lifespan psychology is the change in motivational priorities with age. The *Selection, Optimization and Compensation Model* proposes that with increasing age, people become more selective in their goals. Motivational changes manifest in *selection* and shift towards accessible goals, *optimization* of remaining skills and consequently ensuring *compensation* for deficits and resource losses [11]. In humans, age-related physiological changes in motivation and cognition are difficult to separate from cultural and societal influences, however [12]. Therefore, non-human primates (hereafter ‘primates') are increasingly recognized as important models to identify the processes that underlie behavioural changes with increasing age, as cultural effects and explicit awareness of a limited remaining lifetime can be largely ruled out [13]. The value of primate models lies also in the fact that they undergo similar physiological ageing processes to humans [14].

Several studies have explored changes in primate cognitive ability across age. A classic task to assess inhibitory control in this context is the detour reaching task, where individuals have to reach around a transparent barrier to obtain a food reward [15]. Aged primates in this task appear to have more difficulties in solving this task, which has been attributed to their declining inhibitory capabilities [16]. Considering the entire lifespan, some studies reported a U-shaped distribution indicating that both younger and older primates show difficulties in suppressing prepotent movements. Furthermore, older primates tend to be less flexible in problem-solving behaviour and exhibit difficulties in adapting to changing reward contingencies [17]. As in humans, results in primates studies are inconsistent, ranging from no deficits, to deficits in early adulthood, to severe impairment with age [18].

While age-related cognitive changes in primates have been extensively studied, it is unclear whether motivational changes follow a similar or different trajectory across age. Some studies in primate and non-primate species have already addressed developmental differences in motivation. For instance, in spotted hyenas (*Crocuta crocuta*), and several bird species, juveniles were more persistent and invested more time in food retrieval tasks than adult subjects [19]. Yet, these studies did not assess how motivation changes beyond young adulthood. In primates, there are only a few studies that have dealt with motivational changes in later stages of adulthood, revealing mixed results. A study on chimpanzees (*Pan troglodytes*) found that older chimpanzees tended to be less explorative in problem-solving tasks [20]. Furthermore, a study in Barbary macaques showed that the motivation to explore novel objects declined once individuals reached the reproductive age but was modulated by the availability of a food reward up to an age of about 20 years. Subjects older than that did not engage in the task even when a food reward was available [21]. Our study builds on these previous findings and aims to explicitly compare age-related changes in three processes involved in successful problem-solving, namely inhibitory control, cognitive flexibility and motivation (persistence). Our semi-free-ranging study population is naturally ageing, and monkeys did not receive any training prior to testing. This aspect distinguishes our work from studies in captivity, where primates are typically separated for cognitive testing, which allows them to focus on the task at hand. Moreover, subjects often have a long history of training and testing that promotes the formation of learning sets [17]. Our study thus contributes to a better understanding of how changes in cognition and motivation play out under more natural conditions.

Barbary macaques were presented with different problem-solving tasks, including a detour task to assess inhibitory control, a sliding doors task to test cognitive flexibility and an unsolvable task to assess persistence. We expected to find a U-shaped performance curve, with younger (juvenile and subadult, hereafter ‘young': ≤5 years in females, and ≤7 years in males) and older adults ( >20 years, hereafter ‘old') being impaired in their ability to suppress an impulsive movement, compared with middle-aged monkeys. Regarding the cognitive flexibility task, we expected that with increasing age, monkeys would show a higher latency to start searching for an alternative strategy after experiencing their first choice not being functional. Persistence can involve aspects of inhibitory control as well as cognitive flexibility, since being persistent in a task accounts for trying the same movement or strategy again, but it also means flexibly choosing alternative strategies to achieve a goal. For this reason, we included an unsolvable task to investigate age-related changes in persistence. We expected that with increasing age, monkeys become less persistent and spend less time exploring the task. For all three tasks, we assessed whether the monkeys engaged in the task when confronted with the respective set-up. This gave us another opportunity to gauge motivational changes with age. We predicted that with increasing age, monkeys would be less inclined to engage in the task.

## 2. Methods

### (a) Subjects

Data collection was carried out in a large, age-heterogeneous population of Barbary macaques (*Macaca sylvanus*) ranging at ‘La Forêt des Singes' in Rocamadour, France. Owing to the captive setting, veterinary care and no predation, monkeys in this facility live for up to 30 years. We considered monkeys as ‘old' from 20 years onwards, which is comparable with other macaque studies [21]. The monkeys live in three social groups, comprising a total of 170 (in 2017) to 180 (in 2018) monkeys. They have regular contact with tourists, who can observe and feed them. The monkeys are habituated to behavioural observations. All monkeys are recognizable by their inner-leg tattoo and/or by distinctive physical appearance [22]. The monkeys are provisioned with fruits and vegetables. They also feed on natural vegetation including leaves, grains or insects. Water is provided *ad libitum*. In total, 143 monkeys with an age range from 2 to 30 years participated in at least one and up to all three experimental conditions (see electronic supplementary material for details).

### (b) Apparatuses

**Figure 1.**
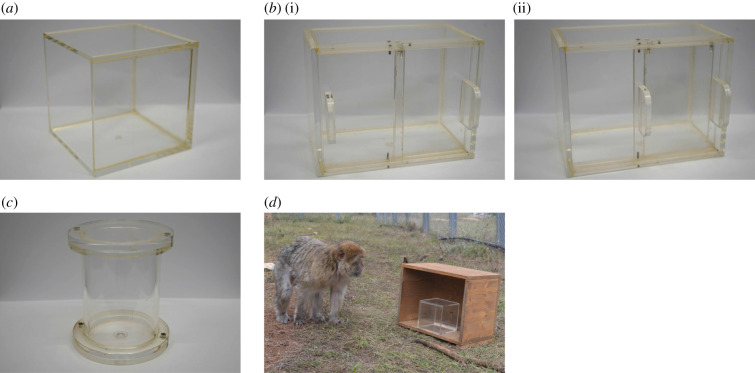
Experimental set-up and apparatuses. Inhibitory control task (*a*); cognitive flexibility (*b*), (i) closed, (ii) left side open; persistence (*c*); set-up of Experiment 1 in the field (*d*).

For the inhibitory control task, a transparent cube (15 × 15 cm^2^, made out of Plexiglas) that was open at one side was fixed in the middle front of a wooden box (50 × 40 × 30 cm, figure 1*a*). The opening of the cube faced either the left or the right side of the wooden box. To retrieve a peanut from the inside, the monkey had to reach around the transparent barrier.

For the cognitive flexibility task, a transparent compartment (20 × 10 × 15 cm, made out of Plexiglas) with two sliding doors was fixed in the middle of a wooden box (50 × 40 × 30 cm, figure 1*b*). The sliding doors could be opened by two handles that were placed at the edge of each sliding door on the left or right side. During testing, one of the sides was blocked by a screw and could be only opened 1 cm, whereas the opposite sliding door could be fully opened. A peanut was placed in the side with the blocked door, on the assumption that the monkeys would try to open this side first and then potentially look for alternative ways to obtain the reward. The opening side (left/right) in the inhibitory control task and the cognitive flexibility task was balanced across sex and age.

For the motivation task, a Plexiglas tube (diameter 10/9 cm × 12 cm) was fixed inside a wooden box (45 × 35 × 25 cm, figure 1*c*). The tube was fixed with two opposing metal nuts that allowed the tube to spin but not to unscrew. The tube could not be opened by the monkeys. Figure 1*d* displays the experimental set-up during a testing session.

### (c) Procedure

We conducted trials only when there was no other monkey in the vicinity (within approx. 10 m) or when the vegetation was dense enough to prevent visual access of nearby monkeys. The wooden box containing one of the three apparatuses was placed by experimenter 1 in such a way that the opening faced the monkey. Experimenter 2 stood at 5 to 10 m from the apparatus at approximately 45° to the apparatus and filmed the trial. Experimenter 2 was visible to the monkey but looking through the display of the camera to avoid eye contact. Experimenter 1 baited the transparent apparatus for both tasks (inhibitory control/cognitive flexibility) with a peanut before the trial started, while turning his/her back to the monkey in order to prevent any visual cues as to how the apparatus could be operated. The transparent tube used for the motivation task remained baited with two peanuts during all trials.

At the beginning of a trial, experimenter 1 showed, and if necessary cracked, a peanut to attract the monkey's attention. This peanut was placed directly in front of the box, which had already been baited. After placing the peanut in front of the box, the experimenter walked away, trying to leave the monkey's sight as quickly as possible. The trial start was defined as the moment when the peanut was placed outside the box. If a monkey stopped exploring for 30 s but then continued, we kept on recording. The trial was terminated when a monkey retrieved the peanut from inside the transparent apparatus for the inhibitory control and cognitive flexibility tasks. Otherwise, the trial ended when the monkey left the test area (2 m radius around the wooden box), when the monkey stayed within the 2 m radius without paying attention for more than 2 min (indicated by looking away or starting to feed/rest), or when another monkey approached. These criteria also determined the end of the motivation task, which was unsolvable. If a monkey had been disturbed, this monkey was tested again in the next field season, ensuring a pause of at least 4 months. In cases where one of the trials had been aborted and the monkey was tested again, we presented the opposing side (i.e. first aborted trial: left side open in the inhibitory control task, second trial: right side open).

Tests were conducted in May, June and September 2016, as well as April, and September to November 2017. In total, we conducted 313 trials involving 143 monkeys (age range: 2 to 30 years old). Sixteen trials had to be discarded either as a result of an experimenter making a experimenter mistake (*N* = 1) or because another monkey interrupted the trial (*N* = 15), resulting in 297 trials used in the analysis. For subjects that participated in more than one task, we aimed to balance the order. We paid attention that the presentation of tasks was balanced regarding age and sex (electronic supplementary material, tables S1–S3).

### (d) Data analysis

We recorded all trials using a Panasonic HC-X929 camera. We measured the exploration time during each trial, which was the time the monkeys actively explored the apparatus until they obtained the peanut or abandoned the task (in the case of the unsolvable motivation task). In the case of the cognitive flexibility task, we also measured the latency from which the monkeys touched the handle on the blocked side until they started exploring the other side's handle at the openable door (time to switching handle).

Because the animal's motion was critical to determine whether it was still manipulating the experimental apparatus, we opted to use a stopwatch to measure exploration time (to the nearest millisecond) rather than coding the videos frame by frame. To assess the accuracy of measurement, we measured the exploration from each video recording three times. A third of the videos were coded by a second rater. We used the intra-class correlation coefficient (ICC (1,*k*)) from the R package *irr* to calculate both intra- and inter-reliability [23]. Intra-reliability for the three measurements for both raters was high: 0.99 (ICC(1,*k*)). For statistical analysis, we used the mean exploration time calculated from the three measurements by the first rater. The inter-rater reliability of the mean was 0.998. Both reliability measurements indicate excellent agreement [24].

### (e) Statistical analysis

For all statistical analysis, we used the R statistical software [25]. We used Generalized Linear Models (GLM), with the *lme4* package [26] to investigate if age influenced the likelihood that the monkeys engaged in the tasks. We further tested if age affected the likelihood of success in the inhibitory control task. We also controlled for sex as a potential influence. Data were fitted to a binomial distribution (engagement with the task yes/no or success yes/no) with a logit link function. Prior to running the models, we tested for heteroscedasticity and overdispersion with the package *DHARMa* [27].

We used Generalized Additive Models for Location, Scale and Shape (GAMLSS) from the *gamlss* package to test for effects of age on exploration time or time of handle switch [28]. We used sex as a control variable owing to potential effects on the exploration time or time of handle switch. GAMLSS provide a framework to model nonlinear relationships and comprise residual heteroscedastic normal regression with effects on the mean and variance. Owing to an assumed lognormal distribution, which does not allow zeros in the dependent variable, we used a subset excluding all individuals that did not explore the apparatus at all. Model diagnostics were based on the residual plots available in the package *gamlss*.

## 3. Results

**Figure 2.**
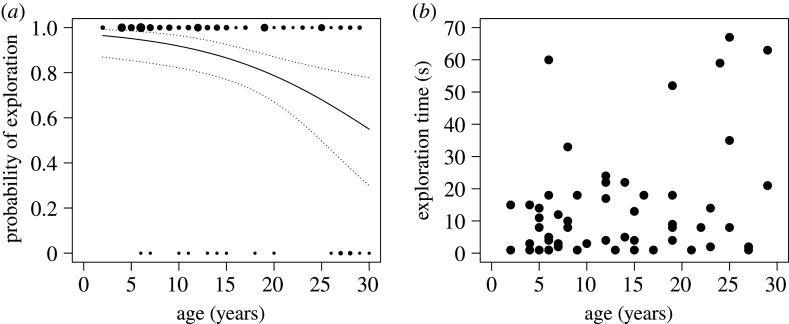
Results of the inhibitory control task. (*a*) Probability of engaging in the task in relation to age. The dots indicate individual engagement in the task (1: yes, 0: no). The dot size corresponds to the number of individuals of the respective age. The solid black line is the regression line of the computed model. The dotted lines indicate the lower (2.5%) and upper (97.5%) confidence intervals of the model. (*b*) Exploration time (s) in relation to age. Each dot represents one individual.

Of the 99 monkeys that were confronted with the inhibitory control task (age: 2 to 30 years), 83 engaged in the task. Older monkeys were less likely to engage in the task than younger ones (GLM: *N* = 99, *z* = −2.86, *p* = 0.004, figure 2*a*). Two monkeys did not enter the 2 m area and did not obtain the peanut that had been placed in front of the apparatus. They were both at the upper end of the age spectrum (26 and 28 years). From the 83 monkeys that engaged in the inhibitory control task, 54 monkeys were successful and retrieved the peanut from inside the cube. Every single monkey that explored the cube tapped against the transparent wall before eventually reaching around. The likelihood of being successful (including also monkeys that did not explore) was not age dependent (GLM: *N* = 99, *z* = −1.52, *p* = 0.13). Excluding the monkeys that did not explore the task, we still did not find an age effect on the likelihood of being successful (GLM: *N* = 54, *z* = −0.05, *p* = 0.96). The unsuccessful monkeys' exploration time did not differ with age (GAMLSS: *N* = 29, *t* = −0.79, *p* = 0.44). Considering the successful monkeys, we did not find the expected U-shaped performance curve. Although some of the older monkeys needed more time to succeed, this effect was not statistically significant (GAMLSS: *N* = 54, *t* = 0.1, *p* = 0.91, figure 2*b*). We also did not find a linear decrease in exploration time (GAMLSS: *N* = 54, *t* = 1.48, *p* = 0.15) and there was no effect of sex on exploration time (GAMLSS: *N* = 54, *t* = 1.1, *p* = 0.29; electronic supplementary material, tables S4–S9).

**Figure 3.**
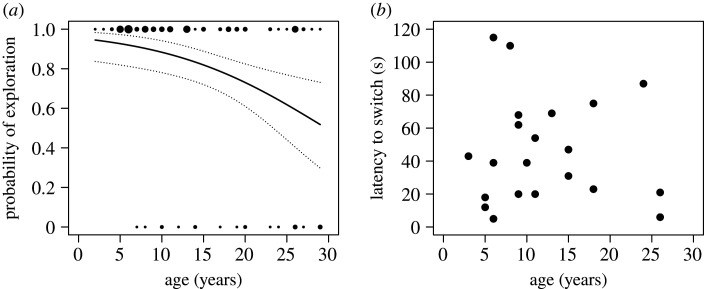
Results of the cognitive flexibility task. (*a*) Probability of engaging in the task in relation to age. The dots indicate individual engagement in the task (1: yes, 0: no). The dot size corresponds to the number of individuals of the respective age. The solid black line is the regression line of the computed model. The dotted lines show the respective lower (2.5%) and upper (97.5%) confidence intervals of the model. (*b*) Latency (s) from inspecting the blocked sliding door until exploring the opposite side in relation to age.

From the 99 individuals presented with the cognitive flexibility task (age: 2 to 29 years), 20 monkeys did not engage in the task. The likelihood of exploring was lower for older monkeys compared with younger ones (GLM: *N* = 99, *z* = −2.98, *p* = 0.003, figure 3*a*). All the monkeys tried to open the blocked side first. One monkey (27 years old) did not approach at all. Of the 79 monkeys that participated, only 21 checked the opposing door of the apparatus. Of these, only nine monkeys managed to open this opposing door and obtained the peanut. Because of the small sample size, we did not analyse the exploration time with regard to success. Considering the overall exploration time, irrespective of success, we did not find that older monkeys explored for a longer time period than younger monkeys (GAMLSS: *N* = 79, *t* = −1.55, *p* = 0.13). We did not find evidence that older monkeys needed a longer latency before they switched to the functional sliding door (GAMLSS: *N* = 21, *t* = −1.51, *p* = 0.15, figure 3*b*). There was no effect of sex on exploration time (GAMLSS: *N* = 21, *t* = 0.39, *p* = 0.7; electronic supplementary material, tables S10–S12).

**Figure 4.**
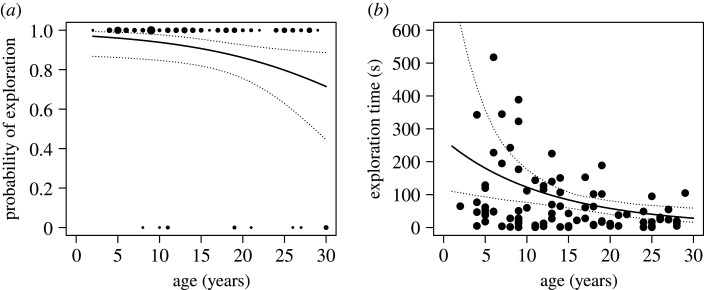
Results of the motivation task. (*a*) Probability of engaging in the task with respect to age. The dots correspond to individual engagement in the task (1: yes, 0: no). The dot size corresponds to the number of individuals of the respective age. The solid black line is the regression line of the computed model. The dotted lines indicate the lower (2.5%) and upper (97.5%) confidence intervals of the model. (*b*) Exploration time (s) in relation to age, with the regression line of the model.

Out of 99 monkeys that were tested in the motivation task (age: 2 to 30 years), 12 did not inspect the transparent tube. The likelihood of engagement in this task was lower for older monkeys compared with younger ones (GLM: *N* = 99, *z* = −2.18, *p* = 0.03, figure 4*a*). Considering the monkeys that explored in the task, older monkeys were more likely to stop turning the baited tube earlier than younger conspecifics (GAMLSS: *N* = 87, *t* = −2.79, *p* = 0.006, figure 4*b*). While one of the younger monkeys (6 years old) explored for around 8 min, all monkeys older than 20 years spent less than 2 min with the tube. There was no clear evidence that sex influenced exploration time (GAMLSS: *N* = 87, *t* = −1.71, *p* = 0.09; electronic supplementary material, tables S13 and S14).

Further, we tested if there was a relationship between the performance in the motivation task and the inhibitory control task. It appears that there was no strong correlation (Spearman rank correlation: *N* subjects = 38, rho = 0.24, *p* = 0.14). Monkeys that were persistent in exploring in the motivation task were neither faster nor slower in succeeding in the inhibitory control task. We did not include the cognitive flexibility task in our analysis here since only eight monkeys succeeded and the sample size for assessing any relationship between the performances in this task compared with the other two was insufficient.

## 4. Discussion

Barbary macaques presented with three experimental set-ups to test inhibitory control, cognitive flexibility and persistence exhibited clear age-related variation in motivation to engage with the tasks and persistence. Although most subjects approached the apparatus to obtain the peanut that had been placed in front of the apparatus, older subjects were less likely to try to obtain the reward inside the apparatus. Furthermore, monkeys older than 20 years spent less time with the unsolvable task than younger monkeys.

Among those monkeys that did engage in the inhibitory control task, we did not find age-related differences in the time nor in the likelihood to succeed. We found appreciable variation in the time and likelihood to succeed between individuals. We observed neither the expected U-shaped distribution, nor a linear or exponential decline with age. Younger and older monkeys did not seem to have greater difficulties in inhibiting their inclination to reach forward compared with monkeys of middle age. Instead, across all ages, there were several subjects who struggled with this task. For successful older subjects, there were no differences in time to success in relation to age compared with younger ones, suggesting that these older monkeys were on a par with younger ones in terms of their cognitive capacities.

The results for the sliding doors task are somewhat difficult to interpret, as only 21 out of 99 monkeys tested switched to the opposite side. Thus, this task appeared to be beyond the capacity of most of the monkeys, irrespective of age. Further, only nine of these 21 monkeys were able to obtain the reward out of the apparatus, corroborating the notion that the task was too difficult. There was no age difference in the latency until the monkeys switched from the blocked door to the non-blocked door. Whether or not the monkeys switched and how long it took them to do so did not appear to be related to age, but owing to the small sample size, we may simply not have been able to pick up an effect if it existed.

Our results suggest that motivation to engage in cognitive testing and to persist in problem-solving decreases with increasing age, while the ability to inhibit a prepotent response or to switch to an alternative strategy is less clearly related to age. To be clear, our results do not allow us to conclude that the cognitive capacity of older monkeys does not decline. Possibly, older subjects that did not participate would have performed poorly. Studies that rely on voluntary participation are unable to resolve this question. Yet, given the results of the inhibitory control task, where a substantial number of older monkeys participated, we assume it is warranted to conclude that motivation and cognitive ability vary independently with age. Overall, motivation seems to follow a different trajectory from inhibitory control and cognitive flexibility. In line with our results, a study on chimpanzees found that aged chimpanzees tended to explore less compared with their younger conspecifics [20]. Neophobia, i.e. the avoidance of novel objects or situations, can also influence an individual's preference for exploration and represents one aspect of the motivational spectrum [29]. A previous study in Barbary macaques revealed that the interest for novel objects seemed to diminish early in adulthood, whereas the presence of a reward boosted the likelihood to explore up into old age.

Changes in activity patterns with increasing age might add to the decrease in motivation to participate and persist. Considering the time spent on highly energetic activities like running, climbing and jumping, a study on Barbary macaques found an age-related decline, suggesting that older monkeys were less physically able or less motivated to engage in such activities [30]. The exploration of the tasks in our study also required physical engagement to obtain the reward. That the older monkeys gave up earlier could thus be associated with avoidance of energetic activities and possibly also physical deterioration. Aged Barbary macaques in a previous study had pronounced difficulties in obtaining a food reward out of a transparent tube. Both sides of the tube were blocked by cotton tissue which needed to be removed to obtain the reward [21]. Task difficulty might have played a role here since success depended largely on the degree of physical prowess.

The loss of motivation in our study population could arise from a similar mechanism of preserving resources with increasing age to that which has been attributed to motivational changes in humans. For older humans, the *Selection, Optimization and Compensation Model* explains how changes in motivation affect goal setting [31]. More specifically, it is assumed that with increasing age achievable goals are preferably selected. Younger adults were more willing to take greater risks in order to achieve a goal and persisted more in improving their performance. By contrast, older adults were instead motivated to counteract losses in cognitively demanding tasks [32]. Further, the perceived effort required for successful performance in cognitive tests was higher for older adults than younger, which ultimately reduced older adults' motivation to participate (effort withdrawal) [33]. Taken together, our findings corroborate the notion that primates and humans show similar changes in motivation in old age.

Further, we expected to find age-associated changes in inhibitory control and cognitive flexibility tasks. In contrast with our predictions, older monkeys did not perform worse than younger ones. Previous work also showed mixed results for an age effect in cognitive performance [18,34]. For example, a study in orang-utans (*Pongo pygmaeus*) showed no age effect (age range 3.5 to 25 years) in a detour reaching and a reversal-learning task where individuals had to flexibly change to an alternative solution once the previously learned strategy was not functioning any more [15]. By contrast, aged common marmosets (*Callithrix jacchus*) displayed more difficulties in inhibiting their movement to reach forward instead of reaching around a barrier, while younger marmosets were all capable of performing successfully in this task [16]. The presence of a food reward could further impact not only the motivation to engage in a task but also the performance during testing. In chimpanzees, the visible presence of a food reward afflicted performance [35]. A similar effect may explain our result in the cognitive flexibility task. The poor performance might have been enhanced by the fact that the door behind which the food item was placed could be moved slightly. This movability of the blocked door potentially triggered the repeated attempts to open it.

The comparison between studies is somewhat hampered by methodological variation. For instance, experience with transparent objects is likely to play a role in dealing with novel problem-solving tasks that include a transparent barrier [36]. We assume that familiarity with transparent objects did not impact the performance in our study however, as the last study involving transparent objects was carried out 2 years before we started [21]. Furthermore, some studies included extensive training sessions before the actual testing started [17]. Such approaches involve learning and memory, which have been shown to exhibit age-associated decline [37]. Older rhesus macaques (*Macaca mulatta*), for example, needed more time to learn the training regime than their younger conspecifics [38]. Moreover, subjects that do not pass specific criteria may be excluded, resulting in a potential overestimation of the species or group-typical abilities. Another factor contributing to motivation and performance is housing conditions, which impact the value of engaging in cognitive testing [21,34]. For instance, a study on rhesus macaques found no age-related decline in the motivation or success in a food retrieval puzzle [34]. In contrast with the Barbary macaque population, which lived in a near-natural setting in a large outdoor enclosure, the rhesus monkeys were housed either alone or in pairs. As there is a complete confound between species and housing conditions, solid conclusions may not be drawn at this stage, but the findings are in line with the idea that housing conditions need to be considered when assessing motivation. Perhaps in more natural settings older subjects prefer to avoid potential conflicts with others over a food reward and therefore refrain from engaging in the task. It may also be possible that they allocate their attention to other aspects in their environment rather than focusing on a puzzle—at least when they are otherwise satiated.

In summary, our experiments with a large, age-heterogeneous group of Barbary macaques revealed greater variation in motivation than cognitive functioning with age, underscoring the importance of considering age-related motivational changes as a key factor in cognitive performance. At the same time, our study highlights the need to carefully control variation in motivation due to reward salience and value, as well as the presence of conspecifics (as competitors or social partners). This is of importance for cross-species comparisons used to reconstruct the evolution of specific abilities. While the application of identical methods in cognitive testing, as implemented in the ‘ManyPrimates' project [39], is an important step in the right direction, motivational variation due to age, prior experience and housing conditions constitutes an important factor to control for in such studies. Overall, our finding that motivation declines with age aligns with the human literature and corroborates the view that insight into a limited future lifetime is not the sole reason for a shift in motivation in old age.

## Supplementary Material

Supplementary Online Material

## Data accessibility

The dataset used in this study is accessible from the Dryad Digital Repository: https://dx.doi.org/10.5061/dryad.kprr4xh12 [40].
