# Supplementary material for: Differential ageing trajectories in motivation, inhibitory control and cognitive flexibility in Barbary macaques (Macaca sylvanus)
Source: Philos Trans R Soc Lond B Biol Sci. 2020 Sep 21;375(1811):20190617. doi: 10.1098/rstb.2019.0617 (PMC7540953; doi:10.1098/rstb.2019.0617)
Supplement: Supplementary Online Material [file rstb20190617supp1.docx]

Supplementary Online Material

**Differential aging trajectories in motivation, inhibitory control and cognitive flexibility in Barbary macaques (*Macaca sylvanus*)**

Eva-Maria Rathke & Julia Fischer

*Correspondence to:* Eva-Maria Rathke (erathke@dpz.eu)

In total, 143 monkeys with an age range from 2 to 30 years participated in at least one and up to all three experimental conditions. In 2016, we tested 32 adult males (≥ 7 years old), 39 adult females (≥ 5 years old), 4 sub-adult males (3-6 years old), one sub-adult female (4 years old), and one juvenile male. In 2017, we tested 47 adult males, 67 adult females, 17 sub-adult males, three sub-adult females, one juvenile male and one juvenile female.

| Table 1 | |
| --- | --- |
| Order of the presentation of all tasks. The numbers indicate if a subject was presented with the inhibitory control, cognitive flexibility or motivation task first, second or third. To illustrate, for 63 monkeys the first task they participated in was the inhibitory control task. | |
| Task order | Number of tests |
| Inhibitory control task | 99 |
| 1 | 63 |
| 2 | 29 |
| 3 | 7 |
| Cognitive flexibility task | 99 |
| 1 | 43 |
| 2 | 44 |
| 3 | 12 |
| Motivation task | 99 |
| 1 | 37 |
| 2 | 38 |
| 3 | 24 |
| Table 2 | |
| Distribution of tasks per age group. | |
| Age group | Number of tests |
| Young (1 to 10 years old) | 104 |
| Inhibitory control task | 36 |
| Cognitive flexibility task | 35 |
| Motivation task | 33 |
| Middle (11 to 20 years old) | 107 |
| Inhibitory control task | 36 |
| Cognitive flexibility task | 34 |
| Motivation task | 37 |
| Old (21 to 30 years old) | 86 |
| Inhibitory control task | 27 |
| Cognitive flexibility task | 30 |
| Motivation task | 29 |

| Table 3 | |
| --- | --- |
| Distribution of tasks per sex. | |
| Sex | Number of tests |
| Females | 150 |
| Inhibitory control task | 51 |
| Cognitive flexibility task | 46 |
| Motivation task | 53 |
| Males | 147 |
| Inhibitory control task | 48 |
| Cognitive flexibility task | 53 |
| Motivation task | 46 |

INHIBITORY CONTROL TASK

| Table 4 | | | | | |
| --- | --- | --- | --- | --- | --- |
| Influence of age and sex on the motivation to engage in the task (yes/no) | | | | | |
| Term | Estimate ± Std. Error | z value | Confidence interval (lower) | Confidence interval (higher) | P |
| (Intercept) | 4.08 ± 0.94 | 4.33 | 2.42 | 6.16 | < 0.001 |
| age | -0.11 ± 0.04 | -2.87 | -0.19 | -0.04 | 0.004 |
| sexmale | -1.16 ± 0.63 | -1.84 | -2.47 | 0.03 | 0.06 |
| The GLM was based on 99 tests (one per subject). | | | | | |

| Table 5 | | | | | |
| --- | --- | --- | --- | --- | --- |
| Influence of age and sex on the success in the task (includes all individuals that were presented with the task, independent of exploration) | | | | | |
| Term | Estimate ± Std. Error | z value | Confidence interval (lower) | Confidence interval (higher) | P |
| (Intercept) | 1.16 ± 0.52 | 2.23 | 0.17 | 2.23 | 0.03 |
| age | -0.04 ± 0.03 | -1.52 | -0.09 | 0.01 | 0.13 |
| sexmale | -0.82 ± 0.43 | -1.93 | -1.68 | 0.00008 | 0.06 |
| The GLM was based on 99 tests (one per subject). | | | | | |

| Table 6 | | | | | |
| --- | --- | --- | --- | --- | --- |
| Influence of age and sex on the success in the task (only including individuals that explored the task) | | | | | |
| Term | Estimate ± Std. Error | z value | Confidence interval (lower) | Confidence interval (higher) | P |
| (Intercept) | 0.92 ± 0.55 | 1.68 | -0.13 | 2.05 | 0.09 |
| age | -0.001 ± 0.03 | -0.05 | -0.06 | 0.06 | 0.96 |
| sexmale | -0.59 ± 0.47 | -1.24 | -1.52 | 0.33 | 0.21 |
| The GLM was based on 54 tests (one per subject). | | | | | |

| Table 7 | | | | | |
| --- | --- | --- | --- | --- | --- |
| Influence of age and sex on exploration time of unsuccessful individuals | | | | | |
| Mu Coefficients | | | | | |
| Term | Estimate ± Std. Error | t value | Confidence interval (lower) | Confidence interval (higher) | P |
| (Intercept) | 1.87 ± 0.69 | 2.73 | 0.53 | 3.22 | 0.01 |
| age | -0.03 ± 0.04 | -0.79 | -0.10 | 0.04 | 0.44 |
| sexmale | 0.57 ± 0.53 | 1.08 | -0.47 | 1.60 | 0.30 |
|  | | | | | |
| Sigma Coefficients | | | | | |
| Term | Estimate ± Std. Error | t value | Confidence interval (lower) | Confidence interval (higher) | P |
| (Intercept) | 0.17 ± 0.36 | 0.48 | -1.17 | 1.52 | 0.63 |
| age | -0.0007 ± 0.023 | -0.03 | -0.07 | 0.07 | 0.98 |
| sexmale | 0.12 ± 0.28 | 0.42 | -0.92 | 1.15 | 0.68 |
| The GAMLSS was based on 29 tests (one per subject). | | | | | |

| Table 8 | | | | | |
| --- | --- | --- | --- | --- | --- |
| Influence of age and sex on exploration time of successful individuals (quadratic model) | | | | | |
| Mu Coefficients | | | | | |
| Term | Estimate ± Std. Error | t value | Confidence interval (lower) | Confidence interval (higher) | P |
| (Intercept) | 1.36 ± 0.59 | 2.3 | 0.2 | 2.51 | 0.03 |
| age | 0.02 ± 0.1 | 0.2 | -0.18 | 0.23 | 0.84 |
| I(age^2) | 0.0004 ± 0.003 | 0.1 | -0.007 | 0.007 | 0.91 |
| sexmale | 0.39 ± 0.36 | 1.1 | -0.32 | 1.09 | 0.29 |
|  | | | | | |
| Sigma Coefficients | | | | | |
| Term | Estimate ± Std. Error | t value | Confidence interval (lower) | Confidence interval (higher) | P |
| (Intercept) | -0.04 ± 0.23 | -0.19 | -1.2 | 1.11 | 0.85 |
| age | 0.01 ± 0.01 | 1.07 | -0.19 | 0.22 | 0.29 |
| sexmale | 0.19 ± 0.22 | 0.89 | -0.51 | 0.90 | 0.38 |
| The GAMLSS was based on 54 tests (one per subject). | | | | | |

| Table 9 | | | | | |
| --- | --- | --- | --- | --- | --- |
| Influence of age and sex on exploration time of successful individuals (linear model) | | | | | |
| Mu Coefficients | | | | | |
| Term | Estimate ± Std. Error | t value | Confidence interval (lower) | Confidence interval (higher) | P |
| (Intercept) | 1.31 ± 0.34 | 3.85 | 0.64 | 1.97 | < 0.001 |
| age | 0.03 ± 0.02 | 1.48 | -0.01 | 0.07 | 0.15 |
| sexmale | 0.38 ± 0.36 | 1.07 | -0.31 | 1.08 | 0.29 |
|  | | | | | |
| Sigma Coefficients | | | | | |
| Term | Estimate ±  Std. Error | t value | Confidence interval (lower) | Confidence interval (higher) | P |
| (Intercept) | -0.05 ± 0.22 | -0.24 | -0.72 | 0.61 | 0.82 |
| age | 0.014 ± 0.01 | 1.13 | -0.03 | 0.06 | 0.26 |
| sexmale | 0.2 ± 0.2 | 1.01 | -0.49 | 0.90 | 0.32 |
| The GAMLSS was based on 54 tests (one per subject). | | | | | |

COGNITIVE FLEXIBILITY

| Table 10 | | | | | |
| --- | --- | --- | --- | --- | --- |
| Influence of age and sex on the motivation to engage in the task (yes/no) | | | | | |
| Term | Estimate ± Std. Error | z value | Confidence interval (lower) | Confidence interval (higher) | P |
| (Intercept) | 2.94 ± 0.75 | 3.91 | 1.58 | 4.57 | < 0.001 |
| age | -0.1 ± 0.03 | -2.98 | -0.17 | -0.04 | 0.003 |
| sexmale | 0.2 ± 0.54 | 0.38 | -0.86 | 1.27 | 0.71 |
| The GLM was based on 99 tests (one per subject). | | | | | |

| Table 11 | | | | | |
| --- | --- | --- | --- | --- | --- |
| Influence of age and sex on exploration time (independent of success) | | | | | |
| Mu Coefficients | | | | | |
| Term | Estimate ± Std. Error | t value | Confidence interval (lower) | Confidence interval (higher) | P |
| (Intercept) | 3.42 ± 0.44 | 7.74 | 2.55 | 4.28 | < 0.001 |
| age | -0.04 ± 0.02 | -1.55 | -0.08 | 0.01 | 0.13 |
| sexmale | -0.16 ± 0.36 | -0.46 | -0.88 | 0.55 | 0.65 |
|  | | | | | |
| Sigma Coefficients | | | | | |
| Term | Estimate ± Std. Error | t value | Confidence interval (lower) | Confidence interval (higher) | P |
| (Intercept) | 0.49 ± 0.19 | 2.57 | -0.37 | 1.36 | 0.01 |
| age | -0.004 ± 0.01 | -0.39 | -0.05 | 0.04 | 0.7 |
| sexmale | 0.001 ± 0.17 | 0.009 | -0.71 | 0.71 | 0.99 |
| The GAMLSS was based on 79 tests (one per subject). | | | | | |

| Table 12 | | | | | |
| --- | --- | --- | --- | --- | --- |
| Influence of age and sex on the latency until the monkey switched from exploring the handle of the blocked side to the opposite side’s handle (openable door) | | | | | |
| Mu Coefficients | | | | | |
| Term | Estimate±  Std. Error | t value | Confidence interval (lower) | Confidence interval (higher) | P |
| (Intercept) | 4.49 ± 0.41 | 10.88 | 3.68 | 5.30 | < 0.001 |
| age | -0.04 ± 0.03 | -1.51 | -0.09 | 0.01 | 0.15 |
| sexmale | 0.14 ± 0.37 | 0.39 | -0.58 | 0.87 | 0.7 |
|  | | | | | |
| Sigma Coefficients | | | | | |
| Term | Estimate ± Std. Error | t value | Confidence interval (lower) | Confidence interval (higher) | P |
| (Intercept) | -0.15 ± 0.34 | -0.44 | -0.96 | 0.66 | 0.67 |
| age | -0.002 ± 0.02 | -0.09 | -0.05 | 0.05 | 0.93 |
| sexmale | -0.15 ± 0.33 | -0.46 | -0.88 | 0.57 | 0.65 |
| The GAMLSS was based on 21 tests (one per subject). | | | | | |

MOTIVATION TASK

| Table 13 | | | | | |
| --- | --- | --- | --- | --- | --- |
| Influence of age and sex on motivation to engage in the task (yes/no) | | | | | |
| Term | Estimate ± Std. Error | z value | Confidence interval (lower) | Confidence interval (higher) | P |
| (Intercept) | 3.19 ± 0.9 | 3.55 | 1.6 | 5.18 | < 0.001 |
| age | -0.09 ± 0.04 | -2.18 | -0.18 | -0.01 | 0.03 |
| sexmale | 0.97 ± 0.72 | 1.35 | -0.36 | 2.54 | 0.18 |
| The GLM was based on 99 tests (one per subject). | | | | | |

| Table 14 | | | | | |
| --- | --- | --- | --- | --- | --- |
| Influence of age and sex on the exploration time | | | | | |
| Mu Coefficients | | | | | |
| Term | Estimate ± Std. Error | t value | Confidence interval (lower) | Confidence interval (higher) | P |
| (Intercept) | 4.51 ± 0.39 | 11.68 | 3.75 | 5.26 | < 0.001 |
| age | -0.06 ± 0.02 | -2.79 | -0.09 | -0.17 | 0.006 |
| sexmale | -0.53 ± 0.31 | -1.71 | -1.14 | 0.08 | 0.09 |
|  | | | | | |
| Sigma Coefficients | | | | | |
| Term | Estimate ±  Std. Error | t value | Confidence interval (lower) | Confidence interval (higher) | P |
| (Intercept) | 0.46 ± 0.22 | 2.09 | -0.3 | 1.21 | 0.04 |
| age | -0.008 ± 0.01 | -0.75 | -0.05 | 0.03 | 0.45 |
| sexmale | 0.08 ± 0.16 | 0.51 | -0.53 | 0.69 | 0.61 |
| The GAMLSS was based on 87 tests (one per subject). | | | | | |
